# Tremorgenesis: a new conceptual scheme using reciprocally innervated circuit of neurons

**DOI:** 10.1186/1479-5876-6-71

**Published:** 2008-11-26

**Authors:** Mario Manto

**Affiliations:** 1 FNRS ULB Erasme, 808 Route de Lennik, 1070 Bruxelles, Belgium

## Abstract

Neural circuits controlling fast movements are inherently unsteady as a result of their reciprocal innervation. This instability is enhanced by increased membrane excitability. Recent studies indicate that the loss of external inhibition is an important factor in the pathogenesis of several tremor disorders such as essential tremor, cerebellar kinetic tremor or parkinsonian tremor. Shaikh and colleagues propose a new conceptual scheme to analyze tremor disorders. Oscillations are simulated by changing the intrinsic membrane properties of burst neurons. The authors use a model neuron of Hodgkin-Huxley type with added hyperpolarization activated cation current (I_h_), low threshold calcium current (I_t_), and GABA/glycine mediated chloride currents. Post-inhibitory rebound is taken into account. The model includes a reciprocally innervated circuit of neurons projecting to pairs of agonist and antagonist muscles. A set of four burst neurons has been simulated: inhibitory agonist, inhibitory antagonist, excitatory agonist, and excitatory antagonist. The model fits well with the known anatomical organization of neural circuits for limb movements in premotor/motor areas, and, interestingly, this model does not require any structural modification in the anatomical organization or connectivity of the constituent neurons. The authors simulate essential tremor when I_h _is increased. Membrane excitability is augmented by up-regulating I_h _and I_t_. A high level of congruence with the recordings made in patients exhibiting essential tremor is reached. These simulations support the hypothesis that increased membrane excitability in potentially unsteady circuits generate oscillations mimicking tremor disorders encountered in daily practice. This new approach opens new perspectives for both the understanding and the treatment of neurological tremor. It provides the rationale for decreasing membrane excitability by acting on a normal ion channel in a context of impaired external inhibition.

## Editorial

Tremor is defined as a rapid oscillation of a body part [1]. Tremor is one of the most common movement disorders encountered in clinical practice and is associated with a neurological disease in most cases [2]. Tremor is distinct from other movement disorders, such as dystonia, chorea, athetosis, tics or myoclonus, even though several movement disorders may co-exist. From a clinical perspective, tremor is classically divided into rest, postural, kinetic and task-specific forms. Action tremor occurs as a result of voluntary contraction of muscles and includes postural, kinetic and isometric tremors. The main disorders associated with these presentations of tremor are given in Table 1. The different pathological tremors are also grouped according to their frequency, amplitude and topographical distribution. Frequencies of pathological tremor in upper limbs range from 3 to 9 Hz in the majority of cases. Tremor disorders are a cause of social difficulties in many patients, impairing numerous activities of daily life. About 25 % of patients do not respond to drugs or neurosurgical therapies. One of the reasons is our lack of understanding in the pathogenesis and natural history of several tremor disorders.

**Table 1:** Main neurological disorders associated with tremor

Type of tremor	Diseases
Rest tremor	Parkinson's disease
	"Parkinson-plus" syndromes
	Drug-induced Parkinsonism
	Stroke
	Post-traumatic tremor
	Psychogenic tremor
Postural tremor	Essential Tremor
	Enhanced Physiological tremor
	Cerebellar ataxias
	Multiple Sclerosis
	Post-traumatic tremor
	Drug-induced postural tremor
	Metabolic diseases
	Psychogenic tremor
Kinetic tremor ("intention tremor")	Cerebellar ataxias
	Essential Tremor
	Multiple Sclerosis
	Psychogenic tremor
Task-specific	Primary writing tremor
	Dystonic tremor
Isometric tremor	Primary and secondary orthostatic tremor*

*Might overlap with essential tremor.

Current theories suggest that tremor is driven by complex combinations of mechanical reflex and central neurogenic oscillations. These oscillations are superimposed on a background of irregular fluctuations in muscle force and limb displacements [3]. In tremors originating in the central nervous system, generators are relatively insensitive to peripheral perturbations in most cases. The mechanical reflex component is dependent upon the inertial and elastic properties of the body [4]. The frequency of passive mechanical oscillations ω depends upon the stiffness K and is inversely related to the inertia I, according to the following equation:

ω = (K/I)^1/2^

**Figure 1:**
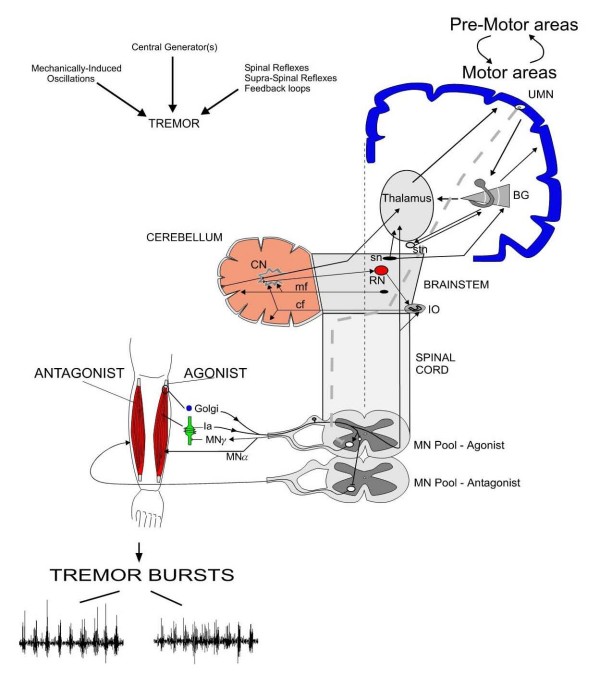
**Illustration of the main anatomical pathways implicated in tremor**. Abbreviations: UMN: upper motor neurons projecting to anterior horn in spinal cord, BG: basal ganglia, stn: subthalamic nucleus, sn: substantia nigra, RN: red nucleus, IO: inferior olivary complex, mf: mossy fibers, cf: climbing fibers, Ia: spindle afferents, MNγ: gamma-motoneuron, MNα: alpha-motoneuron. MN pool: motoneuronal pool.

Several brain areas play a key-role in tremorgenesis (Figure 1). These regions are the main elements of critical loops controlling voluntary and involuntary motor commands. Each of these loops has specific anatomical connections, inherent time delays, adaptable gains and interacts with a myriad of sensory feedback signals [5]:

-the loop between motor cortex and basal ganglia

-the loop between the cerebellum and the brainstem, especially the Guillain-Mollaret triangle, which links dentate nucleus of the cerebellum with the contralateral red nucleus and the inferior olive (this loop is also called the dentate-rubro-olivary tract)

-the loop between the cerebellum, the thalamic nuclei and the motor cortex (cerebello-thalamo-cortical pathway and cortico-ponto-cerebellar tracts)

-the peripheral loops, including the afferences from the muscle spindles to the alpha-motoneurons (spinal loop) and from the peripheral sensors to the motor cortex (transcortical loop). The stretch reflex depends on monosynaptic connections between primary afferent fibers and motor neurons. Spindles also inhibit motor neurons to antagonist muscles through Ia inhibitory interneurons. Afferent fibers from Golgi tendon organs provide a negative feedback for regulating tension via Ib inhibitory interneurons.

Pathological Tremor is usually rhythmic. However, tremor is a non linear and non stationary phenomenon [6]. These last 3 decades, tremor time-series have been mainly analyzed using simultaneous recordings of electromyographic (EMG) activity and acceleration signals, generally measured with piezoresistive accelerometers. Table 2 summarizes the main techniques to assess tremor. Currently, the power spectral analysis is still the most applied tool for neurological disorders manifesting with tremor. Power spectral density (PSD) allows the extraction of the distribution of power. Various parameters indicative of the intensity and variability of tremor are computed, such as centre frequency, frequency dispersion or harmonic index, which help to distinguish pathological tremors [1]. In addition, cross-spectral analysis investigates the interactions and dependencies between several signals, with extraction of phase and coherency spectra. In order to study the roles of specific brain areas in tremor generation, cross-spectral analysis has also been applied between EMG data, electroencephalographic signals (EEG), neuronal discharges in deep brain nuclei, magnetoencephalography (MEG), and other biomedical measurements. Wavelet transforms have also been used effectively for non-stationary signals such as tremor, including for denoising procedures given their advantages as compared to conventional filtering like smoothing. However, all these techniques have shown limits. Current tools have not allowed the grasping of the activity of the brain networks at a cellular level. In addition, there is still a lack of knowledge regarding the neurochemical events occurring at the beginning or throughout the course of tremor disorders. Surprisingly, several drugs currently administered for the management of tremor have been assessed in human in absence of identification of their mechanism of regulation of neuronal discharges related to tremor. For instance, it is unclear how primidone -which is widely administered for essential tremor- affects the neurophysiological and neurochemical properties of brain networks involved in tremor genesis. The effects of the main neurotransmitters implicated (GABA, glutamate, acetylcholine, serotonin, nitric oxide) on the behaviour of central and peripheral oscillators are very complex. This complexity seems even greater when the heterogeneity of the intrinsic properties of each network and the multiple reciprocal connections are taken into account. The translation of the neuronal discharges generated centrally into oscillatory activities in peripheral effectors cannot be understood without attempting to extract the rules governing these elemental neurochemical events. Another factor which has hampered the research in tremor disorders is the difficulty in translating data from animal models, especially from rodent models of tremor [7]. This is the case for instance with the model of acute administration of harmaline in rodents [8], widely used to mimic essential tremor, or for the animal models of Parkinson's disease [9,10]. Despite the fact that 6-hydroxydopamine (6-OHDA) and MPTP (1-methyl-4-phenyl-1,2,3,4-tetrahydropyridine) are very useful for analyzing the mechanisms of dopaminergic neuron degeneration, no remarkable rest tremor similar to parkinsonian tremor is induced by these neurotoxins [7]. They cannot be regarded as a valid model of rest tremor. Trying to isolate mechanisms of tremor from four-footed animals and to extrapolate them to human beings is not straight-forward. Developments of convenient and reproducible methods of evaluation of tremor are needed.

**Table 2:** Clinical and experimental techniques to evaluate tremor

Tool	Parameter analyzed
Clinical scales	Clinical scores of disability
Videos	Clinical characterization of tremor
Quantification of drawings	Evaluation of tremor in 2 dimensions
Surface and needle EMG studies	Assessment of muscle discharges and motor units
Goniometers	Position/displacement
Gyroscopes	Rotational motion
Accelerometers	Acceleration signal
Electromagnetic sensors	Changes in magnetic field
Optoelectronic devices	Position in 3 dimensions
Haptic/Myohaptic devices	Force
Textiles integrating position sensors	Displacement/rotation
Biomechanical modelling	Interactions torques
Neural networks	Simulation of neural circuits

It is currently assumed that most kinds of tremor are associated with an overexcitability of neurons, rendering the neurons prone to discharge in a rhythmic way. Therefore the initial events leading to an increase of excitability deserve attention. Several drugs reducing neuronal membrane excitability improve tremor. This is typically the case with propranolol, GABA-mimetic inhibitory agents such as gabapentin or topiramate, or ethanol. These drugs affect the balance between GABA and glutamate.

In this issue, Shaikh and colleagues propose a new conceptual scheme to analyze tremor disorders [11]. They propose a scheme based on the Sherrington's principle for reciprocal innervation and the phenomenon of post-inhibitory rebound (PIR), which is the rebound increase in firing rates of neurons when the inhibition is removed. These 2 properties render some networks prone to oscillations [12,13]. The authors point out that oscillations in reciprocally innervated circuits appear if the relative effect of intact external inhibition is reduced by an increased excitability within the reciprocally innervated neurons themselves. In other words, increased neural excitability can overcome the effects of normal external inhibition. Increased excitability could result from an increase in either the hyperpolarization activated cation current (I_h_, related to HCN1–HCN4) or the low threshold calcium current (I_t_, related to CaV3 channels) [14,15], or alterations in the intracellular levels of second messengers and the regulators modulating the activation kinetics of these ion channels. Shaikh et al. have tested their hypothesis by simulating a Hodgkin-Huxley type, conductance-based model of pre-motor burst neurons responsible for ballistic limb movements. The authors hypothesize that increased membrane excitability in pre-motor neurons has a key role in pathogenesis of disorders like essential tremor. The circuit consists of reciprocally innervating excitatory neurons and reciprocally inhibiting inhibitory neurons, and includes physiologically-realistic membrane kinetics of the premotor neurons determined by subsets of membrane ion channels. The latter also determines the excitability of the membrane. By increasing specific membrane conductances that are known to increase PIR and neural excitability, such as I_h _and I_t_, they could simulate the range of frequencies of tremor recorded from patients. The increase in these currents resulted in alternating bursts of action potentials in the neurons innervating the sets of agonist and antagonist muscles. The frequency of the simulated tremor was very close to the actual tremor frequency recorded in human.

One of the consequences of this model is the following: interfering with the function of a normal ion channel to decrease membrane excitability in case of impaired external inhibition might reduce the oscillatory behaviour. This might have a special interest for circuits in the thalamus, inferior olive, cerebrum and cerebellum, given their electrophysiological properties and their patterns of innervation. Indeed, these structures are particularly prone to spontaneous or triggered oscillations. Thalamo-cortical firing patterns vary with their membrane potential, and thalamic neurons might behave as oscillators or even resonators [16]. The interaction between cation currents and calcium conductance may generate oscillations from 0.5 to 4 Hz. Animal studies in models of Parkinson's disease suggest that neuronal oscillations are spontaneously generated within the basal ganglia system, especially the pallidum and the subthalamic nucleus, but are mainly synchronized by cortical activity via the striatal inputs. There is an abnormal coupling between the EMG of forearm muscles and the activity in the contralateral primary motor cortex at tremor frequency in this common neurodegenerative disorder [17]. In essential tremor, a bilateral overactivity of cerebellar connections is strongly suspected, with increased synchronous discharges in the olivocerebellar tracts and overall disinhibition of cerebellar nuclei. These latter receive their inputs from the Purkinje cells and are the sole output of the cerebellar circuitry. Predictive computations and rhythmicity in sensorimotor networks are impaired in case of cerebellar lesion [18]. Rhythmicity includes the regular recurrence of events within the information flow, as one can expect in tremor disorders. It is interesting to underline that cerebellar patients present errors in the tuning and timing of activation of agonist and antagonist muscle, as well as motor learning deficits [19,20].

Tremor is attracting the attention of scientists from various disciplines, because of the high prevalence of neurological disorders associated with tremor and thanks to the progress made these last years in terms of better characterization of neurological disorders, mainly with brain imaging (Magnetic Resonance Imaging, Positron Emission Tomography) and molecular biology techniques. The model presented here brings new insights into mechanisms of tremor disorders and also opens direct and short-term perspectives in terms of treatment evaluation. The similarities with the recordings made in patients are outstanding. Furthermore, this model might serve in the future for the deciphering of motor commands and neural representations of movement, the so-called 'internal models' which now encompass not only motor but also cognitive operations [21]. In this sense, this approach would have broader applications in translational medicine.

## References

[B1] Grimaldi G, Manto M (2008). Tremor: from pathogenesis to treatment.

[B2] Bhidayasiri R (2005). Differential diagnosis of common tremor syndromes. Postgr Med J.

[B3] Elble RJ (2003). Characteristics of physiologic tremor in young and elderly adults. Clin Neurophysiol.

[B4] Elble RJ (1996). Central mechanism of tremor. J Clin Neurophysiol.

[B5] Manto M, Bastian AJ (2007). Cerebellum and the deciphering of motor coding. Cerebellum.

[B6] Boose A, Spieker S, Jentgens C, Dichgans J (1996). Wrist tremor: Investigation of agonist-antagonist interaction by means of long-term EMG recording and cross-spectral analysis. Electroencephalogr Clin Neurophysiol – Electromyogr Motor Control.

[B7] Miwa H (2007). Rodent models of tremor. Cerebellum.

[B8] Miwa H, Kubo T, Suzuki A, Kihira T, Kondo T (2006). A species-specific difference in the effects of harmaline on the rodent olivocerebellar system. Brain Res.

[B9] Wang G, Fowler SC (2001). Concurrent quantification of tremor and depression of locomotor activity induced in rats by harmaline and physostigmine. Psychopharmacology (Berl).

[B10] Hattori N, Sato S (2007). Animal models of Parkinson's disease: similarities and differences between the disease and models. Neuropathology.

[B11] Shaikh AG, Kiura K, Optican LM, Ramat S, Tripp RM, Zee DS (2008). Hypothetical membrane mechanisms in essential tremor. J Transl Med.

[B12] Ramat S, Leigh RJ, Zee DS, Optican LM (2005). Ocular oscillations generated by coupling of brainstem excitatory and inhibitory saccadic burst neurons. Exp Brain Res.

[B13] Shaikh AG, Miura K, Optican LM, Ramat S, Leigh RJ, Zee DS (2007). A new familial disease of saccadic oscillations and limb tremor provides clues to mechanisms of common tremor disorders. Brain.

[B14] McCormick DA, Pape HC (1990). Properties of a hyperpolarization-activated cation current and its role in rhythmic oscillation in thalamic relay neurones. J Physiol.

[B15] Shaikh AG, Finlayson PG (2005). Excitability of auditory brainstem neurons, in vivo, is increased by cyclic-AMP. Hear Res.

[B16] Llinás RR, Paré D (1991). Of dreaming and wakefulness. Neuroscience.

[B17] Timmermann L, Gross J, Dirks M, Volkmann J, Freund HJ, Schnitzler A (2003). The cerebral oscillatory network of parkinsonian resting tremor. Brain.

[B18] Molinari M, Leggio MG, Thaut MH (2007). The cerebellum and neural networks for rhythmic sensorimotor representation in the human brain. Cerebellum.

[B19] Diener HC, Dichgans J (1992). Pathophysiology of cerebellar ataxia. Mov Disord.

[B20] Ito M (2000). Mechanisms of motor learning in the cerebellum. Brain Res.

[B21] Kawato M (1999). Internal models for motor control and trajectory planning. Curr Opin Neurobiol.

